# Supplementary material for: The Genetic and Environmental Architecture of the Human Functional Connectome
Source: ArXiv. 2026 Apr 27:arXiv:2604.24614v1. Preprint. [Version 1] (PMC13142550)
Supplement: Supplement 1 [file NIHPP2604.24614v1-supplement-1.pdf]

5 SUPPORTING INFORMATION

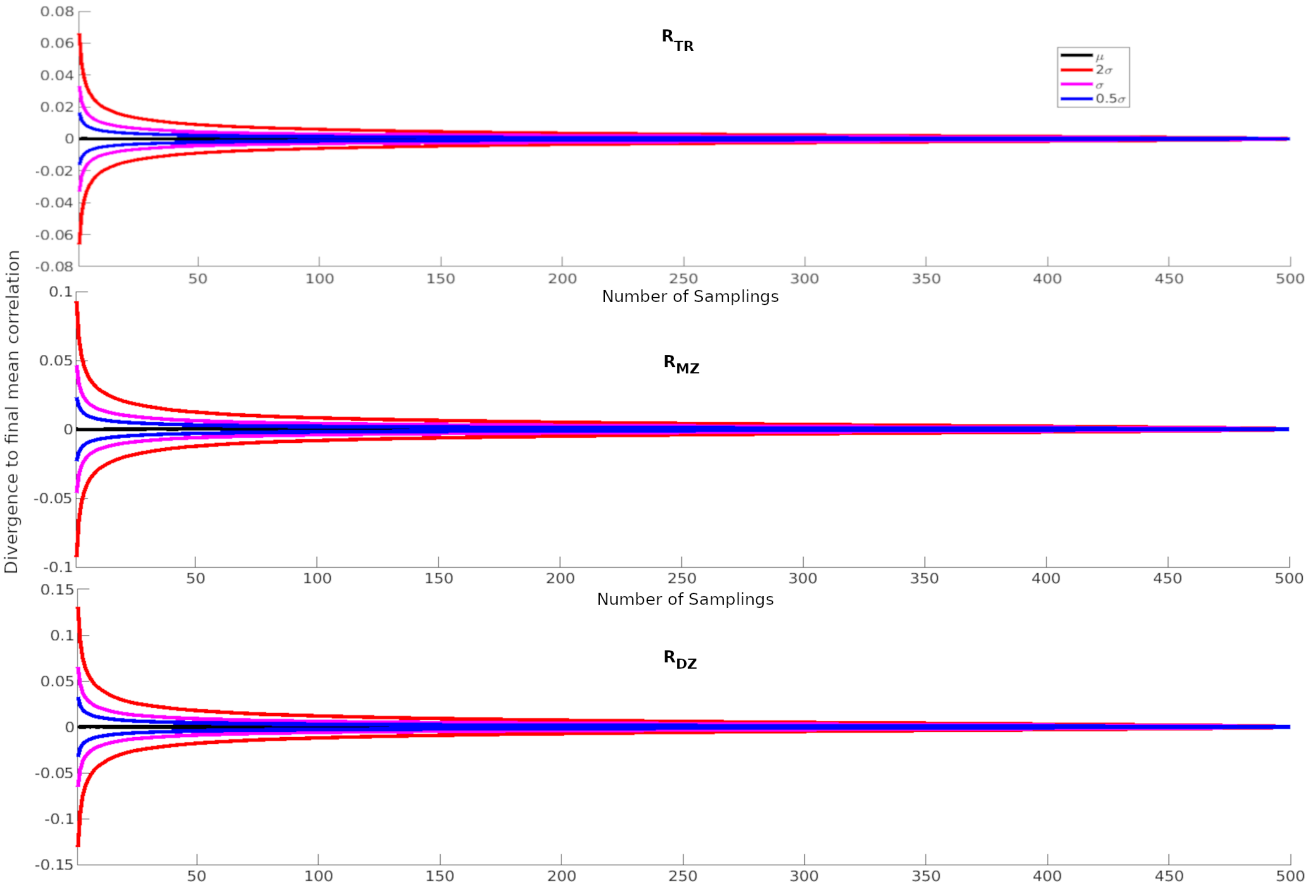

Figure S1: Convergence of correlation estimates across repeated random samplings for test–retest, monozygotic (MZ), and dizygotic (DZ) twin pairs. Each panel shows the divergence of sampled correlation coefficients from the mean correlation as the number of samples increases. The black line represents the mean (μ) across all iterations, while colored lines indicate $\pm 0.5\sigma$ (blue), $\pm\sigma$ (magenta), and $\pm 2\sigma$ (red) bounds. As the number of samplings increases, the variability of the estimates diminishes and the curves converge toward stable mean correlations, demonstrating reliability of the sampling-based correlation estimation procedure.

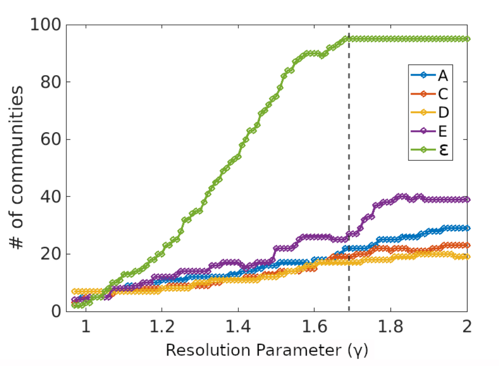

Figure S2: Number of detected communities for each component (A, C, D, E, and $\mathcal{E}$) across the resolution parameter (γ). For the measurement error ($\mathcal{E}$) matrix, the number of communities stabilizes at $\gamma = 1.69$ (vertical dashed line), which we use as the maximum γ value when building the multilayer network. The minimum γ value in the range is chosen as the point where all components first show at least two communities.

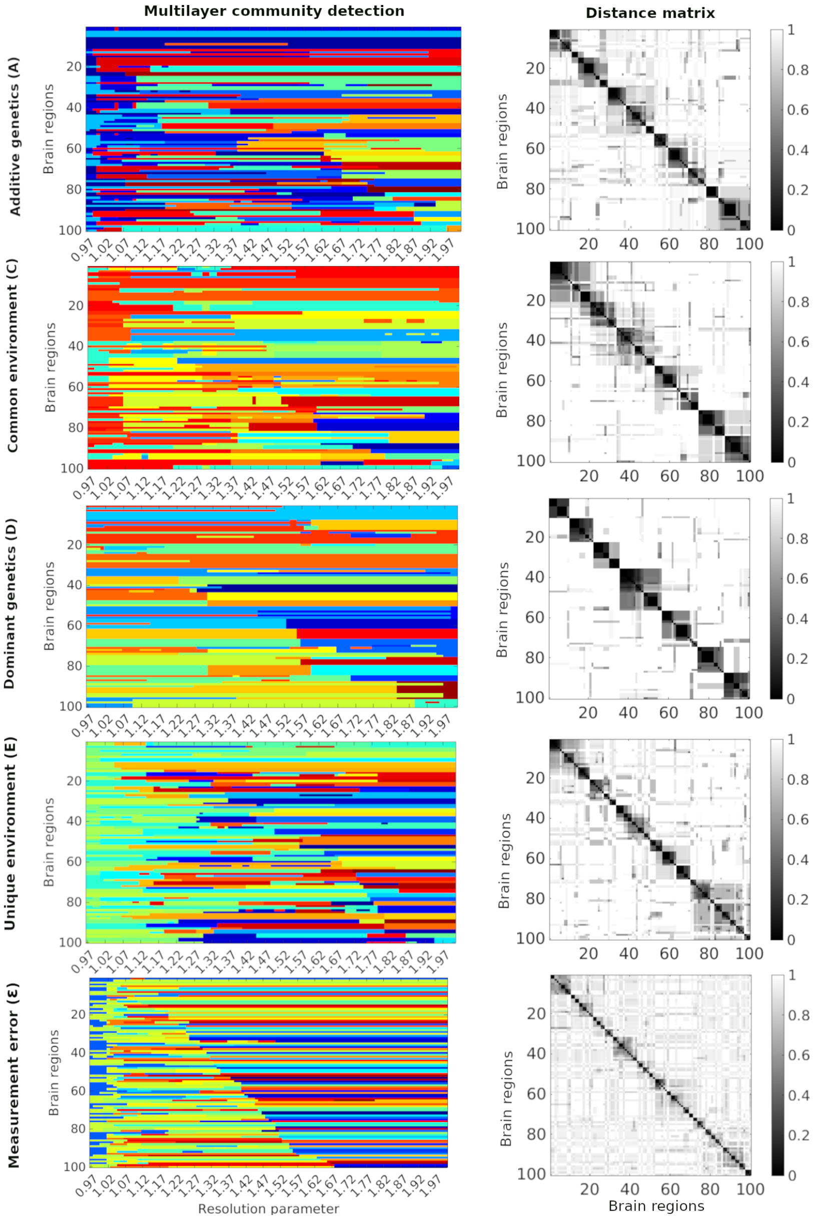

Figure S3: Multilayer community detection for the additive genetics (A), common environment (C), dominant genetics (D), unique environment (E), and measurement error (γ) multiplex networks (left). For each component, brain region community (rows) are shown across the entire γ range, illustrating how modular structure evolves with increasing resolution (columns). The panels on the right display the associated distance matrices, reordered according to hierarchical clustering, which identify sets of regions that exhibit similar modular trajectories across the γ range, revealing the hierarchical organization underlying each component.

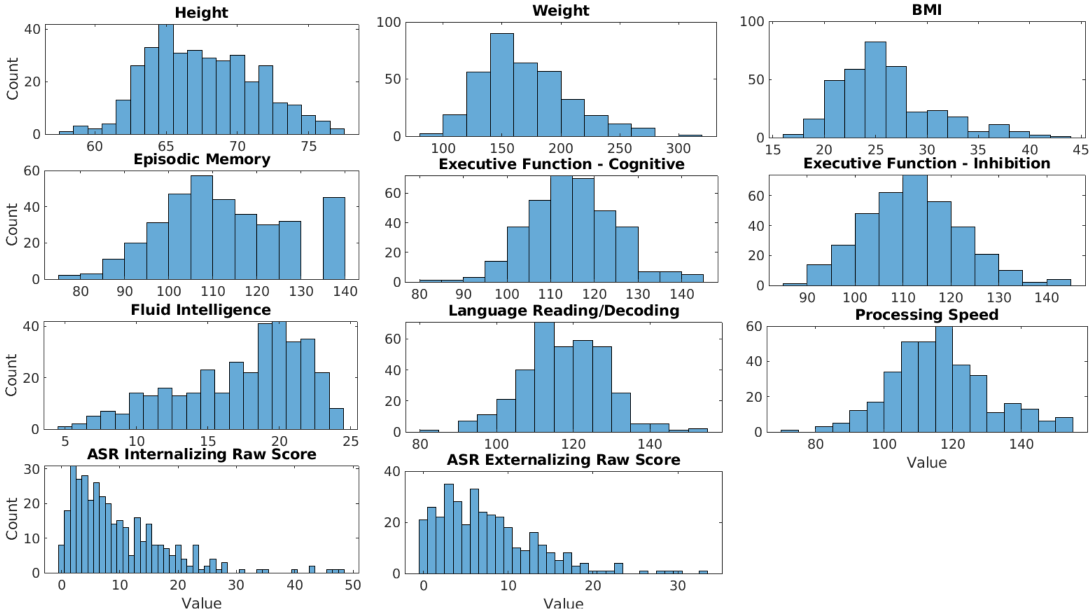

Figure S4: Distribution of demographic, cognitive, and behavioral traits across the full sample. Histograms illustrate the distribution of each trait, including height, weight, BMI, cognitive performance (episodic memory, executive function—cognitive flexibility and inhibition, fluid intelligence, language reading/decoding, and processing speed), and behavioral measures (ASR internalizing and externalizing raw scores).
